# Controversial Areas in Axillary Staging: Are We Following the Guidelines?

**DOI:** 10.1245/s10434-021-10443-x

**Published:** 2021-07-24

**Authors:** Ava Armani, Sasha Douglas, Swati Kulkarni, Anne Wallace, Sarah Blair

**Affiliations:** 1 Department of Surgery, University of California-San Diego, San Diego, CA USA; 2 Department of Surgery, Northwestern University, Chicago, IL USA

## Abstract

**Background:**

Sentinel lymph node biopsy (SLNB) has been the standard of care for clinically node-negative women with invasive breast cancer (IBC); however, there is less agreement on whether to perform SLNB when the risk of metastasis is low or when it does not affect survival or locoregional control.

**Methods:**

An Institutional Review Board-approved survey was sent to members of the American Society of Breast Surgeons asking in which scenarios surgeons would recommend SLNB. Descriptive statistics and multivariable analysis were performed using SPSS software.

**Results:**

There was a 23% response rate; 68% identified as breast surgical oncologists, 6% as surgical oncologists, 24% as general surgeons, and 2% as other. The majority practiced in a community setting (71%) versus an academic setting (29%). In a healthy female with clinical T1N0 hormone receptor-positive (HR+) IBC, 83% favored SLNB if the patient was 75 years of age, versus 35% if the patient was 85 years of age. Academic surgeons were less likely to perform axillary staging in a healthy 75-year-old (odds ratio [OR] 0.51 [0.32–0.80], *p* = 0.004) or a healthy 85-year-old (OR 0.48 [0.31–0.74], *p* = 0.001). For DCIS, 32% endorsed SLNB in women undergoing lumpectomy, with breast surgical oncologists and academic surgeons being less likely to endorse this procedure (OR 0.54 [0.36–0.82], *p* = 0.028; and OR 0.53 [0.34–0.83], *p* = 0.005, respectively).

**Conclusions:**

Despite studies showing that omitting SLNB in older patients with HR+ IBC does not impact regional control or survival, most surgeons are still opting for axillary staging. In addition, one in three are performing SLNB for lumpectomies for DCIS. Breast surgical oncologists and academic surgeons were more likely to be practicing based on recent data and guidelines. Practice patterns are changing but there is still room for improvement.

**Supplementary Information:**

The online version contains supplementary material available at 10.1245/s10434-021-10443-x.

Axillary staging is a critical part of surgery for breast cancer that provides important prognostic information and guides adjuvant treatment recommendations.^1^ Over the last several decades, there has been a shift towards less axillary surgery. Axillary lymph node dissection as the standard of care for every breast cancer patient has long been replaced with sentinel lymph node biopsy (SLNB) for clinically node-negative women with invasive breast cancer (IBC) based on studies showing equivalent survival^2^ and reduced morbidity.^3^ SLNB has also been performed for ductal carcinoma in situ (DCIS), especially in the setting of DCIS with high-risk features.^4^

Nowadays, recommendations for less axillary surgery are shifting towards the omission of SLNB in elderly patients with IBC, where studies have shown that not performing axillary staging has no impact on regional control or survival^5^ and results in improved early quality of life.^6^ Based on these data, the National Comprehensive Cancer Network (NCCN) and the American Society of Breast Surgeons (ASBrS) guidelines indicate that axillary staging should be considered but is not necessary in women over 70 years of age with early-stage hormone receptor-positive (HR+) IBC.^7^–^10^ The Society of Surgical Oncology (SSO) also released the same recommendation in its Choosing Wisely guidelines in 2016, stating “Don’t routinely use sentinel node biopsy in clinically node negative women ≥ 70 years of age with early-stage hormone receptor positive, HER2 negative invasive breast cancer.”^11^ Clinical factors such as tumor grade, stage, and histology can help predict nodal positivity in this population to tailor the omission of SLNB to only the subset of patients with low-risk features in these categories.^12^ Despite these guidelines, there is still debate on when to perform SLNB in this scenario,^13^ and how surgeons have adopted these guidelines is unknown.

Similarly, when to perform SLNB for DCIS is another area of discussion. The NCCN recommends against routine axillary staging in patients with DCIS undergoing breast-conservation surgery (BCS).^7^ However, retrospective reviews of large databases show that axillary staging is often performed and is therefore largely non-compliant with national guidelines. In fact, studies suggest that rates of SLNB for DCIS are increasing in patients undergoing BCS. For patients undergoing mastectomy for DCIS, NCCN guidelines state that SLNB should be considered. Studies again show non-compliance in that a significant portion of these patients are not receiving any axillary evaluation.^14^

The objective of this study was to assess axillary staging practice patterns in controversial scenarios. Specifically, we sought to determine if guidelines for women over 70 years of age with early-stage HR+ IBC were adopted after the Choosing Wisely campaign. We also wanted to evaluate for any changes in practice patterns with DCIS axillary staging.

## Methods

### Survey

A survey questionnaire was developed to evaluate how often surgeons were performing axillary staging in these controversial areas. The survey consisted of 10 scenarios, 4 involving IBC and 6 involving DCIS, for which physicians were asked if they would opt for or against SLNB. Three additional multiple-choice questions assessed if lobular histology, multidisciplinary team influence, or recent changes in guidelines impacted decision making. Sex, specialty, postgraduate training, years in practice, type of practice, and region of practice were also asked of survey respondents. The survey was tested by members of the University of California San Diego (UCSD) breast care team to ensure that it was clear and feasible to complete in a short amount of time. The study was approved by the UCSD Institutional Review Board.

The content and distribution methods of the questionnaire were reviewed and approved by the ASBrS. On 9 January 2020, the ASBrS office sent its members (*n* = 2864) an email with a link to the survey, administered via SurveyMonkey. A reminder email was sent after 3 weeks, and the survey closed after 7 weeks. The methods of data collection were similar to those used for previous ASBrS member surveys on various topics.^15^ The data were collected anonymously.

### Statistics

Survey demographics examined included specialty, sex, practice type, years in training, and region. The effect of each of these variables on each question outcome were analyzed via univariable logistic regression. Those with a *p*-value of < 0.15 were examined in backward model selection multivariable analysis and remained in the final model if the adjusted *p*-value remained at < 0.15. Variables in the final multivariable model with an adjusted *p*-value of < 0.05 were considered significant, and these adjusted *p*-values are reported in the Results section. Spearman’s rank-order correlation was used to determine the relationship between the responses to questions 1–4 of the IBC questions, and questions 1–2 of the DCIS questions, as these should have clear correct responses based on guidelines. This analysis was performed using IBM SPSS Statistics for Macintosh, version 26.0 (IBM Corporation, Armonk, NY, USA).

## Results

Of 2864 active ASBrS members, 666 (23%) responded to the survey, with 625 completing the survey in its entirety. Respondent demographics are detailed in Table 1 and are broken down by specialty (breast surgeon, surgical oncologist, general surgeon, or other), practice type (academic or community), sex (female or male), years in practice (<3, 4–5, 6–10, > 10), and region (northeast or NE, Midwest or MW, South, West, or outside of the US). Each survey question that was asked is reproduced below along with a description of the overall and significant results from multivariable analysis. A more detailed breakdown of the statistical results for each question is provided in the electronic supplementary tables. Based on Spearman’s rank-order correlation, there was a very weak positive correlation between performing SLNB for IBC and DCIS against guideline recommendations (*r*_*s*_ = 0.172, *p* < 0.001).

**Table 1:** Demographics of survey respondents

Variable	*N* (%)
Specialty	
Breast surgeon	426 (68.2)
Surgical oncologist	37 (5.9)
General surgeon	152 (24.3)
Other	10 (1.6)
Practice type	
Academic	184 (29.4)
Community	441 (70.6)
Sex	
Female	429 (68.6)
Male	196 (31.4)
Years in practice	
< 3	74 (11.8)
4–5	49 (7.8)
6–10	93 (14.9)
> 10	409 (65.4)
Region	
Northeast	167 (26.7)
Midwest	135 (21.6)
South	158 (25.3)
West	105 (16.8)
Outside of the US	60 (9.6)

### Invasive Breast Cancer

**Fig. 1:**
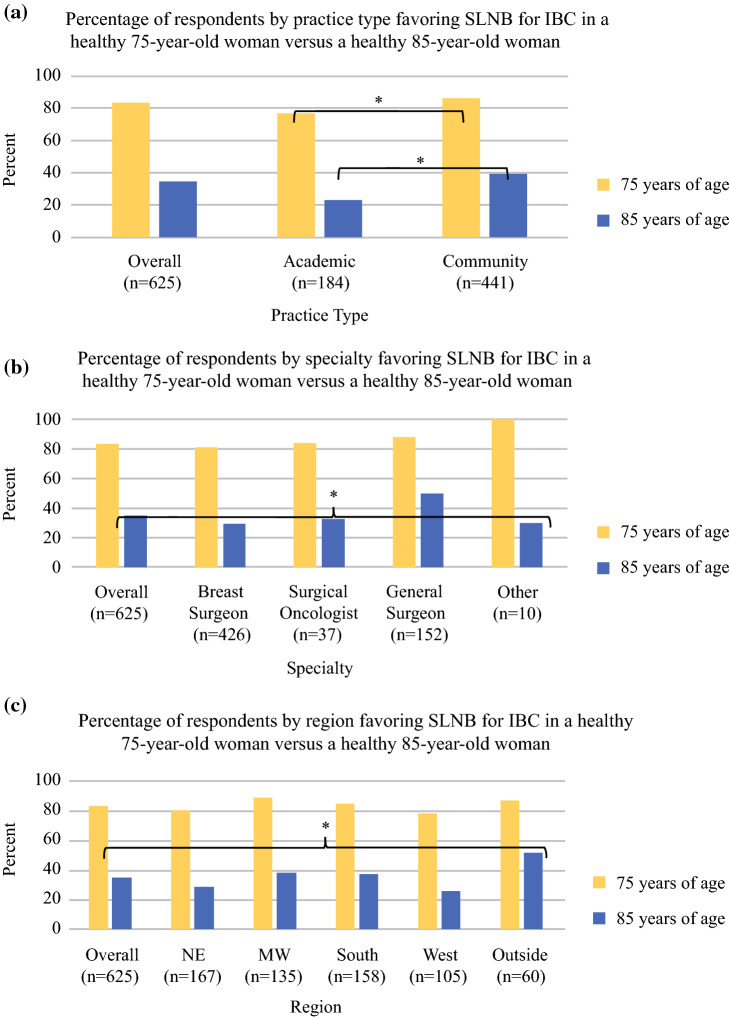
(**a**) Percentage of respondents favoring sentinel lymph node biopsy for invasive breast cancer in a healthy 75-year-old woman compared with a healthy 85-year-old woman, stratified by practice type. Significant differences after multivariable analysis were seen between academic and community centers for both the 75-year-old (*p* = 0.004) and the 85-year-old (*p* = 0.001). (**b**) Percentage of respondents favoring sentinel lymph node biopsy for invasive breast cancer in a healthy 75-year-old women compared with a healthy 85-year-old woman, stratified by specialty. Significant differences after multivariable analysis between specialty response were only seen with regard to the 85-year-old (*p* = 0.005). Note that the *p*-value is for the overall specialty variable and does not distinguish between individual groups. (**c**) Percentage of respondents favoring sentinel lymph node biopsy for invasive breast cancer in a healthy 75-year-old women compared with a healthy 85-year-old woman, stratified by region. Significant differences after multivariable analysis between regional responses were only seen with regard to the 85-year-old (*p* = 0.001). Note that the *p*-value is for the overall region variable and does not distinguish between individual groups. *SLNB* sentinel lymph node biopsy, *IBC* invasive breast cancer

1. *A 75-year-old woman with a 1.9 cm strongly ER/PR+ HER2− invasive ductal carcinoma presents to your office. She is clinically node negative. She has no medical problems. In addition to a lumpectomy, which would you recommend at the time of surgery?*
  In response to this question, 83% of respondents would recommend SLNB, with a significant difference seen between academic and community centers in multivariable analysis (77% vs. 86%, respectively; *p* = 0.004) (Fig. 1a).
2. *An 85-year-old woman with a 1.9 cm strongly ER/PR+ HER2− invasive ductal carcinoma presents to your office. She is clinically node negative. She has no medical problems. In addition to a lumpectomy, which would you recommend at the time of surgery?*
  The percentage of respondents recommending SLNB dropped to 35% for an otherwise healthy 85-year-old with the same tumor as described in question 1. In this scenario, significant variables influencing response included specialty (29% of breast surgeons, 32% of surgical oncologists, 50% of general surgeons, 30% of other; *p* = 0.005), type of practice (23% for academic, 39% for community; *p* = 0.001), and region (26% West, 29% NE, 37% South, 38% MW, 52% outside of the US; *p* = 0.001) (Fig. 1a–c).
3. *A 75-year-old woman with a 1.9 cm strongly ER/PR+ HER2− invasive ductal carcinoma presents to your office. She is clinically node negative. She has a history of diabetes, hypertension and coronary artery disease with cardiac stents placed 6 months ago on ASA 81 mg daily. In addition to a lumpectomy, which would you recommend at the time of surgery?*
  In a 75-year-old woman with multiple comorbidities and the same tumor as the previous two questions, 42% of respondents recommended SLNB. Significant variables correlated with recommending SLNB included specialty (34% of breast surgeons, 43% of surgical oncologists, 63% of general surgeons, 50% of other; *p* < 0.001), practice type (26% academic vs. 48% community), years in training (23% < 3 years, 37% 4–5 years, 45% 6–10 years, 45% > 10 years; *p* = 0.032), and region (36% NE, 47% MW, 49% South, 32% West, 45% outside of the US; *p* = 0.026).
4. *An 85-year-old woman with a 1.9 cm strongly ER/PR+ HER2− invasive ductal carcinoma presents to your office. She is clinically node negative. She has a history of diabetes, hypertension and stage 3 chronic kidney disease not on dialysis. In addition to a lumpectomy, which would you recommend at the time of surgery?*
  For similar comorbidities and tumor characteristics in an 85-year-old, 14% of respondents would recommend an SLNB. Significant variables contributing to this response included specialty (10% of breast surgeons, 11% of surgical oncologists, 25% of general surgeons, and 20% of other; *p* = 0.009), practice type (7% academic, 16% community; *p* = 0.011), years in training (5% < 3 years, 16% 4–5 years, 20% 6–10 years, 13% > 10 years; *p* = 0.028), and region (9% NE, 11% MW, 19% South, 10% West, and 15% outside of the US; *p* = 0.007).
5. *Are you more likely to perform axillary staging on patients with HR+ invasive lobular histology as compared to HR+ invasive ductal histology?*
  Twenty-eight percent responded ‘yes’. Significant variables correlating with this response included sex (31% female, 19% male; *p* = 0.030) and years in training (41% < 3 years, 35% 4–5 years, 36% 6–10 years, 22% > 10 years; *p* = 0.006).
6. *Have you changed your practice regarding the use of sentinel node surgery in women over age 70 in the past 1–3 years?*
  A slight majority (57%) responded ‘yes’. There was a significant difference only in the variable of reported sex, with 63% of female surgeons and 43% of male surgeons responding ‘yes’ (*p* < 0.001).
7. *Does your multi-disciplinary team influence you in adding sentinel node surgery for women over age 70?*

Multidisciplinary teams were reported to play a role in influencing decision to add SLNB for women over age 70 years in 63% of respondents (46% encouraging SLNB, 17% discouraging SLNB).

### DCIS

**Fig. 2:**
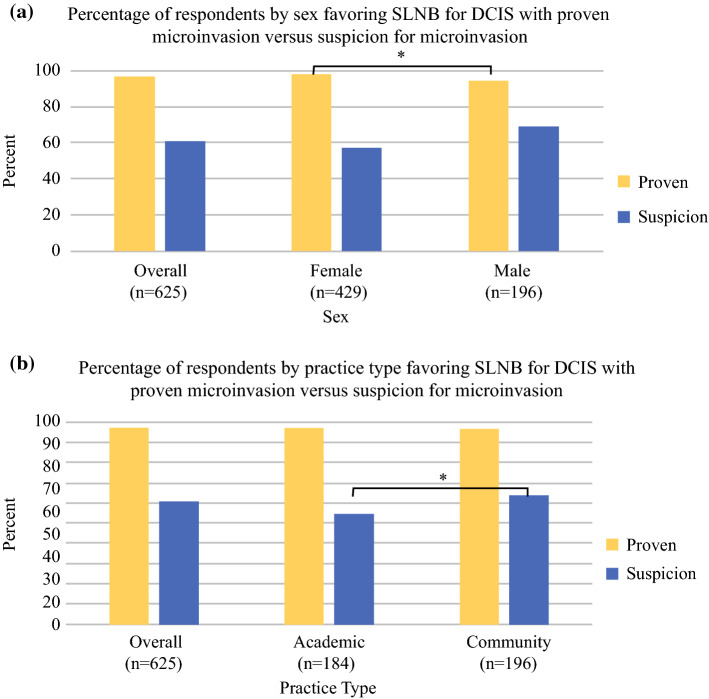
(**a**) Percentage of respondents favoring sentinel lymph node biopsy for ductal carcinoma in situ with proven microinvasion compared with suspicion for microinvasion, stratified by sex. Significant differences after multivariable analysis were only seen between sex with regard to proven microinvasion (*p* = 0.015). (**b**) Percentage of respondents favoring sentinel lymph node biopsy for ductal carcinoma in situ with proven microinvasion compared with suspicion for microinvasion, stratified by practice type. Significant differences after multivariable analysis were only seen between practice type with regard to suspicion for microinvasion (*p* = 0.040). *SLNB* sentinel lymph node biopsy, *IBC* invasive breast cancer

1. *A 55-year-old woman with 5 cm of biopsy proven strongly ER/PR+ DCIS and a very large breast presents to your office. She is clinically node negative. She has no medical problems. In addition to a lumpectomy, which would you recommend at the time of surgery?*
  Thirty-four percent of respondents would recommend adding SLNB. The two variables with significant influence on this decision included practice type (26% academic, 37% community; *p* = 0.009) and years in practice (16% < 3 years, 22% 4–5 years, 28% 6–10 years, 40% > 10 years; *p* < 0.001).
2. *A 45-year-old woman with 3 cm high grade ER/PR− DCIS presents to your office. She is clinically node negative. She has no medical problems. In addition to a lumpectomy, which would you recommend at the time of surgery?*
  In this scenario, 32% of respondents would recommend SLNB. The variables associated with significant influence towards SLNB included specialty (28% breast surgeons, 24% surgical oncology, 47% general surgery, 40% other; *p* = 0.028), practice type (21% academic, 37% community; *p* = 0.005), years in practice (24% < 3 years, 12% 4–5 years, 26% 6–10 years, 37% > 10 years; *p* = 0.002), and region (NE 26%, MW 31%, South 33%, West 32%, outside of the US 46%; *p* = 0.030).
3. *In patients undergoing breast conservation, do you perform sentinel lymph node biopsy for patients with DCIS with proven micro-invasion on core biopsy?*
  The vast majority (97%) recommend adding an SLNB. The only significant variable associated with this was surgeon sex (98% female, 94% male; *p* = 0.015) (Fig. 2a).
4. *In patients undergoing breast conservation, do you perform sentinel lymph node biopsy for patients with DCIS with suspicion for micro-invasion on core biopsy?*
  For the same situation as in question 3, with a suspicion of microinvasion rather than proven, the overall respondent recommendation to add SLNB dropped to 61%. Practice type was the only significant variable affecting this decision (54% academic, 64% community; *p* = 0.040) (Fig. 2b).
5. *A 60-year-old woman with strongly ER/PR+ ductal carcinoma in situ requiring mastectomy presents to your office. In addition to mastectomy, which would you recommend at the time of surgery?*
  Nearly all (98%) respondents recommended SLNB, with no significant difference in response by any variable examined.
6. *A 75-year-old woman with strongly ER/PR+ ductal carcinoma in situ requiring mastectomy presents to your office. In addition to mastectomy, which would you recommend at the time of surgery?*

For the same disease in a 75-year-old woman, the rate of SLNB recommendation was 82%, again with no significant differences between variables.

## Discussion

Studies show that SLNB can be omitted when the risk of axillary metastasis is low or when it has no impact on regional control or survival. These data are incorporated into national guidelines (Table 2) but do not always reflect physician practice patterns. Despite guidelines to the contrary, SLNB is performed for elderly women with early-stage HR+ IBC and DCIS.

**Table 2:** National guidelines on axillary staging

	DCIS	IBC
NCCN	Consider an SLN procedure if performing mastectomy or with excision in an anatomic location compromising the performance of a future SLN procedure	May be considered optional in patients who have particularly favorable tumors, patients for whom the selection of adjuvant systemic and/or radiation therapy is unlikely to be affected, the elderly, or those with serious comorbid conditions
SSO		Do not routinely use sentinel node biopsy in clinically node-negative women ≥70 years of age with early-stage hormone receptor-positive, HER2-negative invasive breast cancer
ASBrS	Those having an initial mastectomy or those for whom breast-conservation surgery may prevent future sentinel node mapping should have a simultaneous SLN biopsy	All patients with a clinically negative axilla for whom axillary staging would provide actionable or relevant information should be offered SLN biopsy

*NCCN* National Comprehensive Cancer Network, *SSO* Society of Surgical Oncology, *ASBrS* American Society of Breast Surgeons, *DCIS* ductal carcinoma in situ, *IBC* invasive breast cancer, *SLN* sentinel lymph node, *HER2* human epidermal growth factor receptor

NCCN, ASBrS, and SSO guidelines advocate against routine SLNB in clinically node-negative women ≥70 years of age with early-stage HR+ IBC;^7^,^ 8^,^ 11^ however, our study shows that 83% of surgeons still recommend SLNB for this type of tumor in a healthy 75-year-old patient. This number dropped significantly for an 85-year-old patient in the same scenario, suggesting that a higher age cut-off is used in clinical practice than in national guidelines for omitting SLNB. In both instances, surgeons in community settings were more likely to perform SLNB. In the case of an 85-year-old patient, surgical oncologists were less likely to offer SLNB compared with general surgeons. If patients presenting with early-stage HR+ IBC had multiple comorbidities, respondents were much less likely to perform SLNB. However, 42% would still recommend SLNB in a 75-year-old patient with multiple comorbidities despite national guidelines using age 70 years as the cut-off. Those practicing in an academic setting, specializing in breast or surgical oncology, and practicing for less amount of time were more likely to omit SLNB in this scenario. Ductal versus lobular histology did not influence the decision to offer SLNB, but multidisciplinary teams did influence the decision by mostly encouraging SLNB. Just over half of respondents indicated they had changed their practice regarding SLNB in the last 3 years. Female surgeons were significantly more likely to have changed their practice, consistent with other studies that have shown female physicians are more likely to adhere to clinical guidelines.^16^

Many factors that could be influencing surgeons to perform SLNB are contributing to surgeon adherence to guidelines. First, surgeons are likely recommending SLNB based on functional status and not age,^17^ and are therefore using a higher age cut-off than 70 years. This would explain why surgeons are much more likely to recommend SLNB in a 75-year-old with or without comorbidities, compared with an 85-year-old. Our study showed that community surgeons, those without fellowship training, and those practicing the longest were least likely to adhere to this guideline, which suggests it may be harder to disseminate guidelines to physicians practicing in community settings who did not receive specialized training. It is also likely more difficult for surgeons who have been practicing for longer periods of time to change their established methods of practice. Better methods for disseminating guidelines and educating these groups may help in increasing acquiescence. Finally, almost half of our respondents indicated that multidisciplinary teams encourage them to perform SLNB in elderly women with early-stage HR+ IBC. Medical and radiation oncologists often use information from SLNB to guide their treatment recommendations. For example, the American Society for Radiation Oncology (ASTRO) uses SLNB in this population to determine when to radiate the axilla and when to offer partial breast radiation.^18^ In contrast, when SSO and ASTRO put forth guidelines together regarding margins for lumpectomy, the guidelines were rapidly adopted.^19^,^ 20^ Therefore, it may be preferable to have future guidelines presented by societies across multidisciplinary specialties to facilitate changes in physician practice. Similarly, medical oncologists use SLNB results to guide adjuvant chemotherapy recommendations. However, the recent RxPonder trial showed no benefit to adding chemotherapy for postmenopausal women with early-stage HR+ IBC with one to three positive nodes and a low oncotype score,^21^ further supporting the omission of SLNB in this population. Patients with higher-grade tumors that are not strongly HR+ and HER2-negative may not be good candidates for de-escalation.

Despite long-standing guidelines advocating against SLNB for DCIS patients undergoing BCS, except in instances that prevent a future SLNB, one in three surgeons still recommend this procedure. Many surgeons continue to justify SLNB for DCIS with high-risk features and it continues to be a topic of debate posted on forums.^22^ As with the IBC scenarios, those practicing in a community setting, those who did not have specialized training, and those who were practicing for a greater amount of time were more likely to perform SLNB in these scenarios. Almost all respondents recommended SLNB with proven microinvasion, and over 80% recommended SLNB for DCIS in the setting of mastectomy, which are practices consistent with guidelines.

As our data show, surgeons are performing SLNB in both IBC and DCIS more than is recommended by national guidelines. Although SLNB is usually well tolerated, it does carry risks, such as chronic pain, decreased sensation, decreased strength, and lymphedema.^23^ Therefore, it is important to increase adherence to these guidelines to prevent overtreatment, a primary goal of the Choosing Wisely campaign. A recent article reporting on a large, prospective database showed that in low-risk patients aged 75–79 years with 2 cm, grade 1–2 IBC (*n* = 465), 5-year breast cancer-specific survival (BCSS) was 96% and was not influenced by lymph node positivity.^24^ These data further support de-escalating axillary staging. It is especially concerning that SLNB in DCIS patients undergoing BCS was reported to be 39.4% in 2011,^14^ which is not significantly different than our finding of one in three surgeons performing SLNB in DCIS patients undergoing BCS in 2020. Although our survey focused on high-risk DCIS as opposed to the study by Mitchell et al., SLNB is still not recommended in these scenarios in national guidelines. This suggests that adherence with guidelines is not an issue of time lag but that there needs to be a focus on educating surgeons about national guidelines in settings that have low compliance. Educating surgeons that the risk of upgrade of DCIS to IBC is 21%, with only 12% of these patients having a positive sentinel lymph node,^25^ may also prove to be beneficial. Even in DCIS with high-risk features, such as large tumor size or palpable lesion, the risk of upgrade is still around 21%.^26^

Our study had several limitations. First, we had a relatively low response rate to our survey, with 23% of ASBrS members responding, but this percentage is similar to other surveys to our membership and was still large enough to adequately power the analysis. Second, questions regarding grade were not included. Since grade is predictive of nodal positivity, it can influence surgeons’ decisions to perform SLNB; however, we felt that including grade would add too many variables to each scenario and decrease our response rate even further by making the survey difficult to complete. Third, because the survey was multiple choice, we do not know the exact reasoning behind surgeons’ decisions for or against SLNB in each scenario. Future studies should focus on why surgeons are not adhering to axillary staging guidelines to help identify and potentially target educational gaps.

## Conclusions

Despite guidelines advocating against routine SLNB for older patients with HR+ IBC, the majority of surgeons are still opting for axillary staging. In addition, one in three are still performing SLNB for lumpectomies for DCIS. General surgeons, surgeons in community settings, and those practicing for a longer period of time were less likely to be practicing based on recent data and guidelines. Better methods of dissemination and education in these settings could help decrease overtreatment in patients who do not benefit from SLNB. In addition, respondents were greatly influenced by multidisciplinary teams, suggesting that putting forth guidelines across specialties could also improve physician adherence. Multidisciplinary de- implementation strategies may be necessary to change practice patterns more expeditiously. Educated clinicians should have nuanced discussions on the risks and benefits of appropriate de-escalation of care.

## Supplementary Information

Below is the link to the electronic supplementary material.

Supplementary file1 (DOCX 65 kb)
