# Supplementary material for: Controversial Areas in Axillary Staging: Are We Following the Guidelines?
Source: Ann Surg Oncol. 2021 Jul 24;28(10):5580–7. doi: 10.1245/s10434-021-10443-x (PMC8418590; doi:10.1245/s10434-021-10443-x)
Supplement: Supplementary file 1 — Supplementary file1 (DOCX 65 kb) [file 10434_2021_10443_MOESM1_ESM.docx]

Supplemental

*Please note, empty cells under multivariable columns indicate that the corresponding variable did not meet significance level of p<0.15 to be included in the final multivariable model.*

*Invasive*

| 1. A 75-year-old woman with a 1.9 cm strongly ER/PR+ Her2- invasive ductal carcinoma presents to your office. She is clinically node negative. She has no medical problems. In addition to a lumpectomy, which would you recommend at the time of surgery? | | | | | | |
| --- | --- | --- | --- | --- | --- | --- |
|  | SLNB | No Axillary | Univariable | | Multivariable | |
|  |  |  | OR (95% CI) | p-value | OR (95% CI) | p-value |
| Specialty  Breast Surgery  Surgical Oncology  General Surgery  Other | 346 (81.2)  31 (83.8)  134 (88.2)  0 (0) | 80 (18)  6 (16.2)  18 (11.8)  10 (100) | Reference  1.20 (0.48, 2.96)  1.72 (0.99, 2.98)  - | 0.051 |  |  |
| Gender  Female  Male | 354 (82.5)  167 (85.2) | 75 (17.5)  29 (14.8) | Reference  1.22 (0.77, 1.95) | 0.399 |  |  |
| Practice Type  Academic  Community | 141 (76.6)  380 (86.2) | 43 (23.4)  61 (13.8) | 0.53 (0.34, 0.81)  Reference | 0.004 | 0.51 (0.32, 0.80)  Reference | 0.004 |
| Years in Training  <3 years  4-5 years  6-10 years  >10 years | 57 (77.0)  40 (81.6)  75 (80.6)  349 (85.3) | 17 (23.0)  9 (18.4)  18 (19.4)  60 (14.7) | 0.58 (0.31, 1.06)  0.76 (0.35, 1.66)  0.72 (0.40, 1.28)  Reference | 0.294 |  |  |
| Region  Northeast  Midwest  South  West  Outside US | 133 (79.6)  120 (88.9)  134 (84.8)  82 (78.1)  52 (86.7) | 34 (20.4)  15 (11.1)  24 (15.2)  23 (21.9)  8 (13.3) | Reference  2.05 (1.06, 3.94)  1.43 (0.80, 2.55)  0.91 (0.50, 1.66)  1.66 (.72, 3.83) | 0.109 | Reference  1.83 (0.94, 3.56)  1.24 (0.69, 2.23)  0.77 (0.42, 1.42)  1.81 (0.78, 4.20) | 0.94 |
| Total | 521 (83.4) | 104 (16.6) |  |  |  |  |

| 2. An 85-year-old woman with a 1.9 cm strongly ER/PR+ Her2- invasive ductal carcinoma presents to your office. She is clinically node negative. She has no medical problems. In addition to a lumpectomy, which would you recommend at the time of surgery? | | | | | | |
| --- | --- | --- | --- | --- | --- | --- |
|  | SLNB | No Axillary | Univariable | | Multivariable | |
|  |  |  | OR (95% CI) | p-value | OR (95% CI) | p-value |
| Specialty  Breast Surgery  Surgical Oncology  General Surgery  Other | 125 (29.3)  12 (32.4)  76 (50.0)  3 (30.0) | 301 (70.0)  25 (67.6)  76 (50.0)  7 (70.0) | Reference  1.16 (0.56, 2.37)  2.41 (1.65, 3.52)  1.03 (0.26, 4.06) | <0.001 | Reference  1.35 (0.63, 2.87)  1.99 (1.32, 3.01)  0.90 (0.22, 3.62) | 0.012 |
| Gender  Female  Male | 132 (30.8)  84 (42.9) | 297 (69.2)  112 (57.1) | Reference  1.69 (1.19, 2.40) | 0.003 |  |  |
| Practice Type  Academic  Community | 43 (23.4)  173 (39.2) | 141 (76.6)  268 (60.8) | 0.47 (0.32, 0.70)  Reference | <0.001 | 0.48 (0.31, 0.74)  Reference | 0.001 |
| Years in Training  <3 years  4-5 years  6-10 years  >10 years | 15 (20.3)  16 (32.7)  38 (40.9)  147 (35.9) | 59 (79.7)  33 (67.3)  55 (59.1)  262 (64.1) | 0.45 (0.25, 0.83)  0.86 (046, 1.62)  1.23 (0.78, 1.95)  Reference | 0.025 | 0.56 (0.30, 1.05)  0.99 (0.51, 1.93)  1.36 (0.84, 2.20)  Reference | 0.110 |
| Region  Northeast  Midwest  South  West  Outside US | 48 (28.7)  51 (37.8)  59 (37.3)  27 (25.7)  31 (51.7) | 119 (71.3)  84 (62.2)  99 (62.7)  78 (74.3)  29 (48.3) | Reference  1.51 (0.93, 2.44)  1.48 (0.93, 2.35)  0.86 (0.50, 1.49)  2.65 (1.44, 4.86) | 0.005 | Reference  1.16 (0.69, 1.94)  1.16 (0.71, 1.88)  0.63 (0.36, 1.13)  2.82 (1.50, 5.33) | 0.001 |
| Total | 216 (34.6) | 409 (65.4) |  |  |  |  |

| 3. A 75-year-old woman with a 1.9 cm strongly ER/PR+ Her2- invasive ductal carcinoma presents to your office. She is clinically node negative. She has a history of diabetes, hypertension and coronary artery disease with cardiac stents placed 6 months ago on ASA 81 mg daily. In addition to a lumpectomy, which would you recommend at the time of surgery? | | | | | | |
| --- | --- | --- | --- | --- | --- | --- |
|  | SLNB | No Axillary | Univariable | | Multivariable | |
|  |  |  | OR (95% CI) | p-value | OR (95% CI) | p-value |
| Specialty  Breast Surgery  Surgical Oncology  General Surgery  Other | 145 (34.0)  16 (43.2)  95 (62.5)  5 (50.0) | 281 (66.0)  21 (56.8)  57 (37.5)  5 (50.0) | Reference  1.48 (0.75, 2.92)  3.23 (2.20, 4.75)  1.94 (0.55, 6.80) | <0.001 | Reference  1.70 (083, 3.49)  2.48 (1.64, 3.76)  1.63 (0.45, 5.89) | <0.001 |
| Gender  Female  Male | 159 (37.1)  102 (52.0) | 270 (62.9)  94 (48.0) | Reference  1.84 (1.31, 2.59) | <0.001 |  |  |
| Practice Type  Academic  Community | 48 (26.1)  213 (48.3) | 136 (73.9)  228 (51.7) | 0.38 (0.26, 0.55)  Reference | <0.001 | 0.44 (0.29, 0.67)  Reference | <0.001 |
| Years in Training  <3 years  4-5 years  6-10 years  >10 years | 17 (23.0)  18 (36.7)  42 (45.2)  184 (45.0) | 57 (77.0)  31 (63.3)  51 (54.8)  225 (55.0) | 0.37 (0.20, 0.65)  0.71 (0.39, 1.31)  1.01 (0.64, 1.58)  Reference | 0.003 | 0.43 (0.23, 0.78)  0.86 (0.45, 1.64)  1.06 (0.66, 1.72)  Reference | 0.032 |
| Region  Northeast  Midwest  South  West  Outside US | 60 (35.9)  63 (46.7)  77 (48.7)  34 (32.4)  27 (45.0) | 107 (64.1)  72 (53.3)  82 (51.3)  71 (67.6)  33 (55.0) | Reference  1.56 (0.98, 2.48)  1.70 (1.09, 2.64)  0.85 (0.51, 1.43)  1.46 (0.80, 2.66) | 0.027 | Reference  1.15 (0.69, 1.89)  1.29 (0.80, 2.08)  0.58 (0.33, 1.01)  1.47 (0.78, 2.78) | 0.026 |
| Total | 261 (41.8) | 364 (58.2) |  |  |  |  |

| 4. An 85-year-old woman with a 1.9 cm strongly ER/PR+ Her2- invasive ductal carcinoma presents to your office. She is clinically node negative. She has a history of diabetes, hypertension and stage 3 chronic kidney disease not on dialysis. In addition to a lumpectomy, which would you recommend at the time of surgery? | | | | | | |
| --- | --- | --- | --- | --- | --- | --- |
|  | SLNB | No Axillary | Univariable | | Multivariable | |
|  |  |  | OR (95% CI) | p-value | OR (95% CI) | p-value |
| Specialty  Breast Surgery  Surgical Oncology  General Surgery  Other | 41 (9.6)  4 (10.8)  38 (25.0)  2 (20.0) | 385 (90.4)  33 (89.2)  114 (75.0)  8 (80.0) | Reference  1.14 (0.38, 3.38)  3.13 (1.92, 5.10)  2.35 (0.48, 11.43) | <0.001 | Reference  1.37 (0.44, 4.31)  2.58 (1.48, 4.50)  2.55, (0.50, 12.98) | 0.009 |
| Gender  Female  Male | 46 (10.7)  39 (19.9) | 383 (89.3) | Reference  2.07 (1.30, 3.29) | 0.002 | Reference  1.55 (0.91, 2.66) | 0.112 |
| Practice Type  Academic  Community | 13 (7.1)  72 (16.3) | 171 (92.9)  369 (83.7) | 0.39 (0.21, 0.72) | 0.001 | 0.42 (0.21, 0.85)  Reference | 0.011 |
| Years in Training  <3 years  4-5 years  6-10 years  >10 years | 4 (5.4)  8 (16.3)  19 (20.4)  54 (13.2) | 70 (94.6)  41 (83.7)  74 (79.6)  355 (86.8) | 0.38 (0.13, 1.07)  1.28 (0.57, 2.88) | 0.030 | 0.58 (0.19, 1.76)  1.85 (0.76, 4.47)  2.21 (1.17, 4.19)  Reference | 0.028 |
| Region  Northeast  Midwest  South  West  Outside US | 15 (9.0)  15 (11.1)  30 (19.0)  10 (9.5)  15 (25.0) | 152 (91.0)  120 (88.9)  128 (81.0)  95 (90.5)  45 (75.0) | Reference  1.27 (0.60, 2.69)  2.38 (1.22, 4.61)  1.07 (0.46, 2.47)  3.38 (1.53, 7.44) | 0.005 | Reference  0.80 (0.36, 1.77)  1.63 (0.81, 3.27)  0.75 (0.31, 1.80)  3.29 (1.41, 7.49) | 0.007 |
| Total | 85 (13.6) | 540 (86.4) |  |  |  |  |

| 5. Are you more likely to perform axillary staging on patients with HR+ invasive lobular histology as compared to HR+ invasive ductal histology? | | | | | | |
| --- | --- | --- | --- | --- | --- | --- |
|  | SLNB | No Axillary | Univariable | | Multivariable | |
|  |  |  | OR (95% CI) | p-value | OR (95% CI) | p-value |
| Specialty  Breast Surgery  Surgical Oncology  General Surgery  Other | 123 (28.9)  7 (18.9)  36 (23.7)  3 (30.0) | 303 (71.1)  30 (81.1)  116 (76.3)  7 (70.0) | Reference  0.58 (0.25, 1.34)  0.77 (0.50, 1.17)  1.06 (0.27, 4.15) | 0.394 |  |  |
| Gender  Female  Male | 132 (30.8)  37 (18.9) | 297 (69.2)  159 (81.1) | Reference  0.54 (0.35, 0.79) | 0.002 | Reference  0.63 (0.41, 0.96) | 0.030 |
| Practice Type  Academic  Community | 43 (23.4)  126 (28.6) | 141 (76.6)  315 (71.4) | 0.76 (0.51, 1.14)  Reference | 0.182 | 0.69 (0.46, 1.04)  Reference | 0.071 |
| Years in Training  <3 years  4-5 years  6-10 years  >10 years | 30 (40.5)  17 (34.7)  33 (35.5)  89 (21.8) | 44 (59.5)  32 (65.3)  60 (64.5)  320 (78.2) | 2.45 (1.46, 4.12)  1.91 (1.01, 3.60)  1.98 (1.22, 3.21)  Reference | 0.001 | 2.26 (1.32, 3.86)  1.77 (0.93, 3.39)  1.81 (1.20, 2.97)  Reference | 0.006 |
| Region  Northeast  Midwest  South  West  Outside US | 46 (27.5)  47 (34.8)  123 (77.8)  75 (71.4)  49 (81.7) | 46 (27.5)  47 (34.8)  35 (22.2)  30 (28.6)  11 (18.3) | Reference  1.41 (0.86, 2.29)  0.75 (0.45, 1.24)  1.05 (0.61, 1.81)  0.59 (0.28, 1.23) | 0.073 |  |  |
| Total | 169 (27.5) | 456 (73.0) |  |  |  |  |

| 6. Have you changed your practice regarding the use of sentinel node surgery in women over age 70 in the past 1-3 years? | | | | | | |
| --- | --- | --- | --- | --- | --- | --- |
|  | SLNB | No Axillary | Univariable | | Multivariable | |
|  |  |  | OR (95% CI) | p-value | OR (95% CI) | p-value |
| Specialty  Breast Surgery  Surgical Oncology  General Surgery  Other | 254 (59.6)  21 (56.8)  74 (48.7)  5 (50.0) | 172 (40.4)  16 (43.2)  78 (51.3)  5 (50.0) | Reference  0.76 (0.35, 1.49)  1.13 (0.57, 2.22)  0.72 (0.35, 1.49) | 0.132 |  |  |
| Gender  Female  Male | 269 (62.7)  85 (43.4) | 160 (37.3)  111 (56.6) | Reference  0.46 (0.32, 0.64) | <0.001 | Reference  0.46 (0.32, 0.64) | <0.001 |
| Practice Type  Academic  Community | 110 (59.8)  244 (55.3) | 74 (40.2)  197 (44.7) | 1.20 (0.85, 1.70)  Reference | 0.305 |  |  |
| Years in Training  <3 years  4-5 years  6-10 years  >10 years | 50 (67.6)  28 (57.1)  58 (62.4)  218 (53.3) | 24 (32.4)  21 (42.9)  35 (37.6)  191 (46.7) | 1.83 (1.08, 3.08)  1.17 (0.64, 2.13)  1.45 (0.91, 2.31)  Reference | 0.078 |  |  |
| Region  Northeast  Midwest  South  West  Outside US | 97 (58.1)  81 (60.0)  85 (53.8)  66 (62.9)  25 (41.7) | 70 (41.9)  54 (40.0)  73 (46.2)  39 (37.1)  35 (58.3) | Reference  1.08 (0.68, 1.72)  0.84 (0.54, 1.30)  1.22 (0.74, 2.02)  0.52 (0.28, 0.94) | 0.079 |  |  |
| Total | 354 (56.6) | 271 (43.4) |  |  |  |  |

| 7. Does your multi-disciplinary team influence you in adding sentinel node surgery for women over age 70? | |
| --- | --- |
| Response | Number (%) |
| Yes, encourage | 295 (47.2) |
| Yes, discourage | 109 (17.4) |
| No | 221 (35.4) |

*DCIS*

| 1. A 55-year-old woman with 5 cm of biopsy proven strongly ER/PR+ DCIS and a very large breast presents to your office. She is clinically node negative. She has no medical problems. In addition to a lumpectomy, which would you recommend at the time of surgery? | | | | | | |
| --- | --- | --- | --- | --- | --- | --- |
|  | SLNB | No Axillary | Univariable | | Multivariable | |
|  |  |  | OR (95% CI) | p-value | OR (95% CI) | p-value |
| Specialty  Breast Surgery  Surgical Oncology  General Surgery  Other | 135 (31.7)  13 (35.1)  59 (38.8)  5 (50.0) | 291 (68.3)  24 (64.9)  93 (61.2)  5 (50.0) | Reference  1.17 (0.58, 2.36)  1.37 (0.93, 2.01)  2.16 (0.61, 7.57) | 0.300 |  |  |
| Gender  Female  Male | 138 (32.2)  74 (37.8) | 291 (67.8)  122 (62.2) | Reference  1.28 (0.90, 1.82) | 0.173 |  |  |
| Practice Type  Academic  Community | 48 (26.1)  164 (37.2) | 136 (73.9)  277 (62.8) | 0.60 (0.41, 0.87)  Reference | 0.007 | 0.59 (0.39, 0.88)  Reference | 0.009 |
| Years in Training  <3 years  4-5 years  6-10 years  >10 years | 12 (16.2)  11 (22.4)  26 (28.0)  163 (39.9) | 62 (83.8)  38 (77.6)  67 (72.0)  246 (60.1) | 0.29 (0.15, 0.56)  0.44 (0.22, 0.88)  0.59 (0.36, 0.96)  Reference | <0.001 | 0.32 (0.17, 0.62)  0.45 (0.22, 0.91)  0.61 (0.37, 1.00)  Reference | <0.001 |
| Region  Northeast  Midwest  South  West  Outside US | 49 (29.3)  47 (34.8)  47 (29.7)  40 (38.1)  29 (48.3) | 118 (70.7)  88 (65.2)  111 (70.3)  65 (61.9)  31 (51.7) | Reference  1.29 (0.79, 2.09)  1.02 (0.63, 1.64)  1.48 (0.89, 2.48)  2.25 (1.23, 4.13) | 0.061 | Reference  1.28 (0.77, 2.11)  0.96 (0.59, 1.57)  1.30 (0.76, 2.21)  2.32 (1.24, 4.34) | 0.062 |
| Total | 212 (33.9) | 413 (66.1) |  |  |  |  |

| 2. A 45-year-old woman with 3 cm high grade ER/PR- DCIS presents to your office. She is clinically node negative. She has no medical problems. In addition to a lumpectomy, which would you recommend at the time of surgery? | | | | | | |
| --- | --- | --- | --- | --- | --- | --- |
|  | SLNB | No Axillary | Univariable | | Multivariable | |
|  |  |  | OR (95% CI) | p-value | OR (95% CI) | p-value |
| Specialty  Breast Surgery  Surgical Oncology  General Surgery  Other | 117 (27.5)  9 (24.3)  71 (46.7)  4 (40.0) | 309 (72.5)  28 (75.7)  81 (53.3)  6 (60.0) | Reference  0.85 (0.39, 1.85)  2.32 (1.58, 3.40)  1.76 (0.49, 6.35) | <0.001 | Reference  0.86 (0.38, 1.95)  1.85 (1.22, 2.80)  1.61 (0.44, 5.94) | 0.028 |
| Gender  Female  Male | 122 (28.4)  79 (40.3) | 307 (71.6)  117 (59.7) | Reference  1.70 (1.19, 2.42) | 0.004 |  |  |
| Practice Type  Academic  Community | 39 (21.2)  162 (36.7) | 145 (78.8)  279 (63.3) | 0.46 (0.31, 0.69)  Reference | <0.001 | 0.53 (0.34, 0.83)  Reference | 0.005 |
| Years in Training  <3 years  4-5 years  6-10 years  >10 years | 18 (24.3)  6 (12.2)  24 (25.8)  153 (37.4) | 56 (75.7)  43 (87.8)  69 (75.2)  256 (62.6) | 0.54 (0.31, 0.95)  0.23 (0.10, 0.56)  0.58 (0.35, 0.97)  Reference | <0.001 | 0.66 (0.37, 1.20)  0.25 (0.10, 0.61)  0.60 (0.36, 0.83)  Reference | 0.002 |
| Region  Northeast  Midwest  South  West  Outside US | 44 (26.3)  42 (31.1)  53 (33.5)  34 (32.4)  28 (46.7) | 123 (73.7)  93 (68.9)  105 (66.5)  71 (67.6)  32 (53.3) | Reference  1.26 (0.77, 2.08)  1.41 (0.88, 2.27)  1.34 (0.78, 2.28)  2.45 (1.32, 4.52) | 0.080 | Reference  1.05 (0.61, 1.79)  1.21 (0.73, 2.01)  1.06 (0.61, 1.86)  2.78 (1.45, 5.32) | 0.030 |
| Total | 201 (32.2) | 424 (67.8) |  |  |  |  |

| 3. In patients undergoing breast conservation, do you perform sentinel lymph node biopsy for patients with DCIS with *proven* micro-invasion on core biopsy? | | | | | | |
| --- | --- | --- | --- | --- | --- | --- |
|  | SLNB | No Axillary | Univariable | | Multivariable | |
|  |  |  | OR (95% CI) | p-value | OR (95% CI) | p-value |
| Specialty  Breast Surgery  Surgical Oncology  General Surgery  Other | 414 (97.2)  36 (97.3)  146 (96.1)  10 (100.0) | 12 (2.8)  1 (2.7)  6 (3.9)  0 (0) | Reference  1.04 (0.13, 8.26)  0.71 (0.26, 1.91)  - | 0.778 |  |  |
| Gender  Female  Male | 421 (98.1)  185 (94.4) | 8 (1.9)  11 (5.6) | Reference  0.32 (0.13, 0.81) | 0.015 | Reference  0.32 (0.13, 0.81) | 0.015 |
| Practice Type  Academic  Community | 179 (97.3)  427 (96.8) | 5 (2.7)  14 (3.2) | 1.17 (0.42, 3.31)  Reference | 0.759 |  |  |
| Years in Training  <3 years  4-5 years  6-10 years  >10 years | 70 (94.6)  48 (98.0)  91 (97.8)  397 (97.1) | 4 (5.4)  1 (2.0)  2 (2.2)  12 (2.9) | 0.53 (0.17, 1.69)  1.45 (0.19, 11.41)  1.38 (0.30, 6.25)  Reference | 0.653 |  |  |
| Region  Northeast  Midwest  South  West  Outside US | 165 (97.0)  131 (97.0)  152 (96.2)  101 (96.2)  57 (95.0) | 2 (1.2)  4 (3.0)  6 (3.8)  4 (3.8)  3 (5.0) | Reference  0.40 (0.07, 2.20)  0.31 (0.06, 1.55)  0.31 (0.06, 1.70)  0.23 (0.04, 1.41) | 0.459 |  |  |
| Total | 606 (97.0) | 19 (3.0) |  |  |  |  |

| 4. In patients undergoing breast conservation, do you perform sentinel lymph node biopsy for patients with DCIS with *suspicion* for micro-invasion on core biopsy? | | | | | | |
| --- | --- | --- | --- | --- | --- | --- |
|  | SLNB | No Axillary | Univariable | | Multivariable | |
|  |  |  | OR (95% CI) | p-value | OR (95% CI) | p-value |
| Specialty  Breast Surgery  Surgical Oncology  General Surgery  Other | 251 (58.9)  22 (59.5)  104 (68.4)  3 (30.0) | 175 (41.1)  15 (40.5)  48 (31.6)  7. (70.0) | Reference  1.02 (0.52, 2.03)  1.51 (1.02, 2.24)  0.30 (0.80, 1.17) | 0.039 | Reference  1.06 (0.52, 2.16)  1.30 (0.85, 1.98)  0.28 (0.07, 1.10) | 0.124 |
| Gender  Female  Male | 245 (57.1)  135 (68.9) | 184 (42.9)  61 (31.1) | Reference  1.66 (1.16, 2.38) | 0.005 |  |  |
| Practice Type  Academic  Community | 100 (54.3)  280 (63.5) | 84 (45.7)  161 (36.5) | 0.69 (0.48, 0.97)  Reference | 0.034 | 0.67 (0.45, 0.98) Reference | 0.040 |
| Years in Training  <3 years  4-5 years  6-10 years  >10 years | 41 (55.4)  22 (44.9)  53 (57.0)  264 (64.5) | 33 (44.6)  27 (55.1)  40 (43.0)  145 (35.5) | 0.68 (0.41, 1.13)  0.45 (0.25, 0.81)  0.73 (0.46, 1.15)  Reference | 0.030 | 0.80 (0.47, 1.34)  0.45 (0.24, 0.84)  0.78 (0.49, 1.24)  Reference | 0.072 |
| Region  Northeast  Midwest  South  West  Outside US | 98 (58.7)  80 (59.3)  99 (62.7)  58 (55.2)  45 (75.0) | 69 (41.3)  55 (40.7)  59 (37.3)  47 (44.8)  15 (25.0) | Reference  1.02 (0.65, 1.62)  1.18 (0.76, 1.85)  0.87 (0.53, 1.42)  2.11 (1.09, 4.09) | 0.115 | Reference  0.96 (0.59, 1.56)  1.10 (0.69, 1.75)  0.76 (0.46, 1.27)  2.23 (1.13, 4.39) | 0.050 |
| Total | 380 (60.8) | 245 (39.2) |  |  |  |  |

| 5. A 60-year-old woman with strongly ER/PR+ ductal carcinoma in situ requiring mastectomy presents to your office. In addition to mastectomy, which would you recommend at the time of surgery? | | | | | | |
| --- | --- | --- | --- | --- | --- | --- |
|  | SLNB | No Axillary | Univariable | | Multivariable | |
|  |  |  | OR (95% CI) | p-value | OR (95% CI) | p-value |
| Specialty  Breast Surgery  Surgical Oncology  General Surgery  Other | 421 (98.8)  36 (97.3)  148 (97.4)  10 (100.0) | 5 (1.2)  1 (2.7)  4 (2.6)  0 (0) | Reference  0.43 (0.05, 3.76)  0.44 (0.12, 1.66)  - | 0.580 |  |  |
| Gender  Female  Male | 424 (98.8)  191 (97.4) | 5 (1.2)  5 (2.6) | Reference  0.45 (0.13, 1.57) | 0.217 |  |  |
| Practice Type  Academic  Community | 182 (98.9)  433 (98.2) | 2 (1.1)  8 (1.8) | 1.68 (0.35, 7.99)  Reference | 0.493 |  |  |
| Years in Training  <3 years  4-5 years  6-10 years  >10 years | 74 (100.0)  48 (98.0)  93 (100.0)  400 (97.8) | 0 (0.0)  1 (2.0)  0 (0.0)  9 (2.2) | -  1.08 (0.13, 8.71)  -  Reference | 0.099 | -  1.08 (0.13, 8.71)  -  Reference | 0.099 |
| Region  Northeast  Midwest  South  West  Outside US | 164 (98.2)  132 (97.8)  157 (99.4)  104 (99.0)  58 (96.7) | 3 (1.8)  3 (2.2)  1 (0.6)  1 (1.0)  2 (3.3) | Reference  0.81 (0.16, 4.05)  2.87 (0.30, 27.90)  1.90 (0.20, 18.53)  0.53 (0.09, 3.26) | 0.600 |  |  |
| Total | 615 (98.4) | 10 (1.6) |  |  |  |  |

| 6. A 75-year-old woman with strongly ER/PR+ ductal carcinoma in situ requiring mastectomy presents to your office. In addition to mastectomy, which would you recommend at the time of surgery? | | | | | | |
| --- | --- | --- | --- | --- | --- | --- |
|  | SLNB | No Axillary | Univariable | | Multivariable | |
|  |  |  | OR (95% CI) | p-value | OR (95% CI) | p-value |
| Specialty  Breast Surgery  Surgical Oncology  General Surgery  Other | 355 (83.3)  33 (89.2)  118 (77.6)  7 (70.0) | 71 (16.7)  4 (10.8)  34 (22.4)  3 (30.0) | Reference  1.65 (0.57, 4.80)  0.69 (0.44, 1.10)  0.47 (0.12, 1.85) | 0.196 |  |  |
| Gender  Female  Male | 351 (81.8)  162 (82.7) | 78 (18.2)  34 (17.3) | Reference  1.06 (0.68, 1.65) | 0.800 |  |  |
| Practice Type  Academic  Community | 151 (82.1)  362 (82.1) | 33 (17.9)  79 (17.9) | 1.00 (0.64, 1.56)  Reference | 0.995 |  |  |
| Years in Training  <3 years  4-5 years  6-10 years  >10 years | 60 (81.1)  42 (85.7)  75 (80.6)  336 (82.2) | 14 (18.9)  7 (14.3)  18 (19.4)  73 (1.78) | 0.93 (0.49, 1.76)  1.30 (0.56, 3.02)  0.91 (0.51, 1.61)  Reference | 0.886 |  |  |
| Region  Northeast  Midwest  South  West  Outside US | 135 (80.8)  110 (81.5)  135 (85.4)  83 (79.0)  50 (83.3) | 32 (19.2)  25 (18.5)  23 (14.6)  22 (21.0)  10 (16.7) | Reference  1.04 (0.58, 1.86)  1.40 (0.77, 2.50)  0.89 (0.49, 1.64)  1.19 (0.54, 2.59) | 0.703 |  |  |
| Total | 513 (82.1) | 112 (17.9) |  |  |  |  |
